# Engaging students with clickers in language lessons

**DOI:** 10.1186/2193-1801-3-S1-O8

**Published:** 2014-12-04

**Authors:** Janet, Man-wai Cheung, Alan, Nga-lun Wong, Emmy, Yin-ha Chan

**Affiliations:** Centre for Learning and Teaching, Vocational Training Council, Kragujevac, Hong Kong; Language Centre, Hong Kong Institute of Vocational Education (Lee Wai Lee), Kragujevac, Hong Kong

## Background

To engage students, researchers have been integrating clickers in classroom delivery for study. Clickers, also known as ‘personal response systems’[1], ‘student response systems (SRS), audience response systems (ARS), or personal response systems (PRS)’,[2] are a technology that allow students to respond to the teacher in real time and receive instant feedback from the teacher, thus enhancing learning and teaching[3]. Featuring instance and interaction, the technology can cater to students’ needs and learning styles as ‘[Net Generations] are used to interactive, participatory, investigative enquiry’[3]. Some researchers have explored the functions and effectiveness of clickers for use as question aids in class[4], promotion of active learning[1, 2] and improvement in learning[5]. This study aimed to investigate the effect of clickers as a pedagogical approach on student satisfaction.

## Methods

A web app with the clicker function was integrated into the teaching of English and Communication at Foundation Diploma Level in IVE (LWL), wherever found suitable, as a pedagogical method to engage learners for one semester in one of the classes in which three English modules were delivered. The three modules included one listening and speaking module and two reading and writing modules. The two classes involved in this research were taught by the same teacher. The same sets of TLP were used in the 2 classes, but the difference was in the delivery of selected activities during class: adoption of clickers versus non-technology-aided activities. The teacher would explain answers based on the instant results from the clickers. The corresponding exercises would be replaced by other activities such as discussion in the other class. With model answers, the questions were set in the form of multiple choice or short questions. At the end of the semester, the two classes were invited to do a perception survey and interview.

## Results

Students reflected that using clickers motivated them to learn in lessons (mean: 3.64), supported their learning (mean: 3.72) and reflected the learning progress to the teacher (mean: 3.74), and these values were higher than those in the class using non-technology-aided activities (reported means of 3.33, 3.71 and 3.71, respectively). Seventy-two per cent of the students indicated their interest in future lessons with clicker technology.

As challenges, 26% reflected that they were distracted by the mobile technology, which is 5% higher than that in the other class. Failed and incomplete clicker attempts were recorded in all of the clicker activities due to a weak Wi-Fi connection. The average rate of incompletion of clicker activities was around 43%.

## Conclusions

In general, the positivity in students’ feedback showed the favorability of clickers as a learning tool. In future, clicker questioning techniques, effective ways of embedding clickers in teaching, and their relation with active learning and learning effectiveness can be areas for further study.
